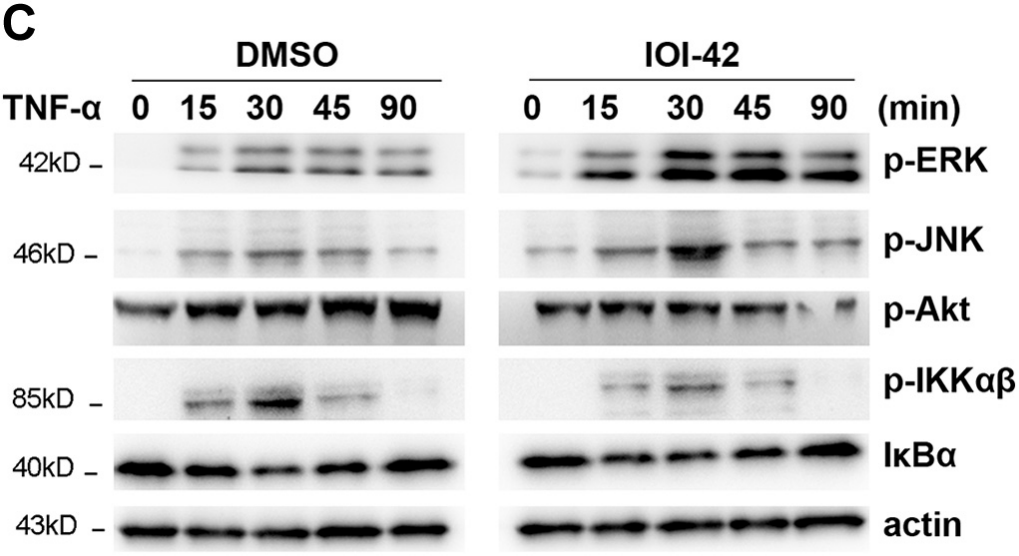# Correction: Potentiation of tumor necrosis factor-α-induced tumor cell apoptosis by a small molecule inhibitor for anti-apoptotic protein hPEBP4

**DOI:** 10.1016/j.jbc.2021.101261

**Published:** 2021-10-06

**Authors:** Jianming Qiu, Jianfeng Xiao, Chaofeng Han, Nan Li, Xu Shen, Hualiang Jiang, Xuetao Cao

The IgG lanes of immunoblot panels Raf-1 and MEK-1 under DMSO in Fig. 6*B* contained blurred pixels. Both 90 minute timepoint bands in the p-IKK⍺/β immunoblots of Fig. 6*C* contained blurred pixels. The authors have provided new versions of the figures from replicate data. The corrections will not affect the results and scientific conclusions of the article.

**Figure 6:**
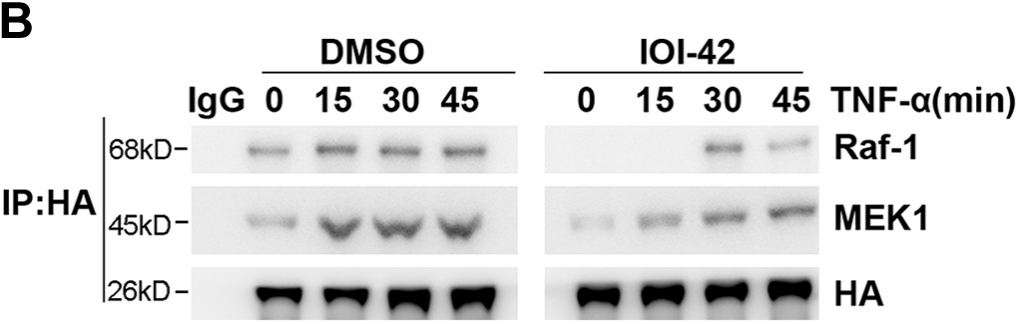

**Figure 6:**